# Officers of the College of Physicians of Philadelphia

**Published:** 1842-08-06

**Authors:** 


*Officers of the College of Physicians of Philadelphia.*

Chosen July 5th, 1842.

*President.*—THOMAS T. HEWSON.

*Vice President.*—John C. Otto.

*Censors.*—Henry Neill, Charles D. Meigs, George B. Wood, J. Wilson Moore.

*Secretary.*—Henry Bond.

*Treasurer.*—J. Rodman Paul.

